# Discriminating Fake From True Brain Injury Using Latency of Left Frontal Neural Responses During Old/New Memory Recognition

**DOI:** 10.3389/fnins.2019.00988

**Published:** 2019-09-25

**Authors:** Jennifer Neal, Stephanie Strothkamp, Esias Bedingar, Patrick Cordero, Benjamin Wagner, Victoria Vagnini, Yang Jiang

**Affiliations:** ^1^Department of Behavioral Science, University of Kentucky College of Medicine, Lexington, KY, United States; ^2^Harvard T.H. Chan School of Public Health, Boston, MA, United States; ^3^Louisville VA Medical Center, Louisville, KY, United States

**Keywords:** malinger, event-related potentials, EEG, traumatic brain injury, P3, late positive component, fractional peak latency

## Abstract

Traumatic brain injury (TBI) is a major public health concern that affects 69 million individuals each year worldwide. Neuropsychologists report that up to 40% of individuals undergoing evaluations for TBI may be malingering neurocognitive deficits for a compensatory reward. The memory recognition test of malingering detection is effective but can be coached behaviorally. There is great need to develop a novel neural based method for discriminating fake from true brain injury. Here we test the hypothesis that decision making of faking memory deficits prolongs frontal neural responses. We applied an advanced method measuring decision latency in milliseconds for discriminating true TBI from malingerers who fake brain injury. To test this hypothesis, latencies of memory-related brain potentials were compared among true patients with moderate or severe TBI, and healthy age-matched individuals who were assigned either to be honest or faking memory deficit. Scalp signals of electroencephalography (EEG) were recorded with a 32-channel cap during an Old/New memory recognition task in three age- and education-matched groups: honest (*n* = 12), malingering (*n* = 15), and brain injured (*n* = 14) individuals. Bilateral fractional latencies of late positive ERP at frontal sites were compared among the three groups under both studied (Old) and non-studied (New) memory recognition conditions. Results show a significant difference between the fractional latencies of the late positive component during recognition of studied items in malingerers (averaged latencies = 396 ms) and the true brain injured subjects (mean = 312 ms) in the frontal sites. Only malingers showed asymmetrical frontal activity compared to the two other groups. These new findings support the hypothesis that that additional frontal processing of malingering individuals is measurably different from those of actual patients with brain injury. In contrast to our previous reported method using difference waves of amplitudes at frontal to posterior midline sites during new items recognition (Vagnini et al., 2008), there was no significant latency difference among groups during recognition of New items. The current method using delayed left frontal neural responses during studied items reached sensitivity of 80% and specificity of 79% in detecting malingers from true brain injury.

## Introduction

Traumatic brain injuries (TBIs) are attributed to 30% of all injury deaths (Taylor et al., 2017) and affect up to 69 million individuals each year worldwide (Dewan et al., 2018). People affected by TBI can suffer from the deficits of their injury for the rest of their life, potentially causing impaired memory, sensation, thinking, movement, and mood swings (Gerberding and Binder, 2003). As severe a health concern as TBI is, it is estimated by neuropsychologists that up to 40% of individuals undergoing evaluations following TBI may be malingering deficits in order to gain a compensatory reward (Mittenberg et al., 2002). Often, those who malinger, or exaggerate symptoms of TBI can be identified by intentional poor performance on cognition tests. Studies have found that, in test seeking compensation, those with mild TBI often exhibited poorer effort and worse cognitive performance than those with moderate or severe TBI (Green et al., 2001). Uncertainty in the legitimacy of the deficits of many patients affected by TBI points to a need for development of a test to screen TBI individuals to validate their deficits, while identifying malingerers.

In recent years, many studies have attempted to find effective methods to distinguish malingering behavior. Sollman and Berry (2011) conducted a large meta-analysis of detection of inadequate effort in neurophysiological testing, which included a group of 21 studies testing memory malingering. Another study has indicated the possibility that those who malinger memory deficits can be identified by the measurable physiological differences of pupil dilatation (Heaver and Hutton, 2011). Research has pointed to identification of malingerers through results of individuals undergoing new and unique testing methods based on subject performance or data processing (McBride et al., 2011; Liu et al., 2016). A classic study examined the measurement of response latency to identify malingerers when undergoing the Portland Digit Recognition Test. Using response latency, researchers were able to successfully classify 74% of malingerers (Rose et al., 1995). The success of this study implicates mental processing time and neural latencies in detection of malingerers. The methodology of using event-related potential (ERP) data to clinically differentiate malingers from those with TBI is fairly rare. If an effective method to distinguish malingering behavior from those with TBI is found, healthcare professionals will be better prepared to treat patients with the appropriate level of care. The Test of Memory Malingering (TOMM) for malingering detection is effective (Tombaugh, 1996; Kanser et al., 2019), but can be coached behaviorally. Finding new methods of identifying malingerers is a significant area of research that holds promise for the healthcare community.

Using combined methods of ERP and reaction time (RT), Vagnini et al. (2008) developed neural and behavioral methods to identify malingerers from TBI patients. The electrophysiological activity was collected using an electroencephalography (EEG) cap during. The TOMM task is a computerized method to test a subject’s memory of images shown to them. Stimulus pictures were shown on a computer screen about 65 cm from the subject. The images themselves were 8 by 10 cm on a white background with a black border. The TOMM task is able to distinguish those who feign memory impairment from those with legitimate memory impairment. If a subject’s score on the TOMM task is low, it suggests an exaggeration of memory impairment symptoms (Tombaugh, 1996).

Event-related potential data are averaged EEG signals that are useful for memory task analysis because the memory recognition events were time-locked to studied (Old) and New items (Finnigan, 2002). Mean ERP amplitudes for malingerers appeared to be reduced compared to those of honest or TBI subjects. Research has documented the abnormalities of ERP data within EEG signals of those with TBI. Certain character of ERP markers is linked to TBI that impact upon many cognitive functions, including processing speed, sustained attention, performance monitoring, inhibitory control, and cognitive flexibility (Dockree and Robertson, 2011). A significant component to this particular event within the EEG signal is the P3 component. The P3 component correlates to decision making and cognition when presented with a stimulus (Patel et al., 2005). The P3a component has been found to have potential to differentiate between those with TBI and those who malinger. Motivations or overt performances to feign brain injury cannot change the character of the P3a component to match that of brain injured individuals, which sets malingerers apart (Hoover et al., 2014).

In using the convenient sample, group differences were compared using advanced fractional latency methods to test a new hypothesis that decision-making of a faker needs additional frontal processing (Tombaugh, 1997). Vagnini et al. (2008) paper utilized complicated analysis of amplitudes of frontal to posterior midline electrodes, while this study focused on latency analysis of lateralized frontal electrodes not previously examined. In comparing the latencies of each subject group, significant differences in neural processing speed can be identified and attributed to the intention to malinger deficits. In contrast to combining RTs and amplitudes of multiple midline electrodes of differences waveforms, latency specific results reveal delayed decision of MNCD could indicate significant markers to identify malingering individuals.

Here we further developed a method measuring latency of neural responses in milliseconds for discriminating true TBI from malingerers who fake brain injury. We test the hypothesis that decision-making of faking memory deficits at each visual item prolongs neural responses during memory recognition.

## Materials and Methods

### Participants

The behavioral and EEG data were collected from 47 age- and sex-matched individuals, which were approved by the medical IRB in the University of Kentucky. The control group was healthy, honest participants (HON) with no history of brain injury instructed to perform the task honestly to the best of their ability (mean age = 36.2; *n* = 16). The second group (MAL) was healthy individuals with no history of brain injury instructed to malinger deficits of TBI while undergoing the task (mean age = 32.7; *n* = 16). The final group consisted of patients with reported TBI instructed to perform the task honestly to the best of their ability (mean age = 40.5; *n* = 15) (Vagnini et al., 2008). Two participants from the TBI, one from the malingering, and four from the honest group were excluded due to excessive artifacts of EEG signals. Frontal, lateralized electrodes have more muscle artifacts compared to those at the midline electrodes. The TBI individuals ranged from moderate to severe TBI. Medical records indicate that the TBI group had a mean emergency room Glasgow Coma Scale score of 8.7 (SD = 2.9), a mean duration of loss of consciousness of 7.2 days (SD = 12.0), were an average of 13 years post-injury (SD = 7.2), and the majority (73%) were injured in moving vehicle accidents. CT and MRI scans indicated brain injury in varied locations from the brain stem, frontal, temporal, occipital, and parietal in both the left and right hemisphere (Vagnini et al., 2008).

### Procedure

The study employed a 32-electrode EEG cap on subjects while undergoing the Old/New Memory Task. Participants’ performances (accuracy and reaction times) were recorded along with EEG scalp signals. Data were recorded using Neuroscan 4.5 and analyzed using EP Toolkit 2.0. This was done by comparing the results from detecting MNCD to the results of the established testing method (TOMM-C) (Tombaugh, 1996, 1997; Vagnini et al., 2008; Kanser et al., 2019).

### Task

**FIGURE 1:**
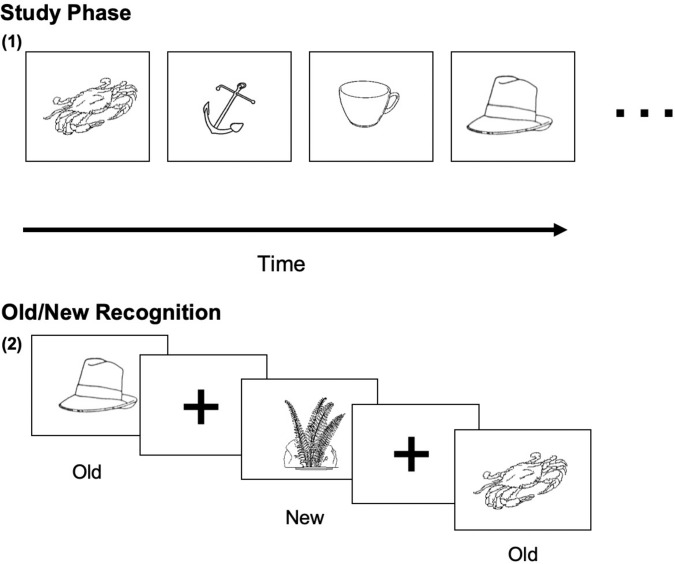
Sample visual stimuli of the Old/New Task. **(1)** The studied (Old) visual object image (5 s each); **(2)** Old/New decision on studied and new (non-studied) images.

The Old/New task began with a study phase of 100 New drawings. Stimulus pictures were displayed on a computer screen, which were presented for 5 s each during the study phase, and participants were instructed to memorize each picture. After a short break, all 100 pictures were studied again for a second time. After studying the pictures, the participants entered the test phase. Participants viewed 140 pictures, presented one time (70 old and 70 foils not yet presented to the participant). For each picture, the participant decided whether the drawing was “New” or “Old” and clicked a corresponding key on the keyboard (Figure 1).

### Data Analysis

Standard EEG preprocessing were performed (e.g., removing artifacts). They have been reported in detail previously (Vagnini et al., 2008). Here, we picked the largest ERP components, P3 or late positive component for latency analysis. The research done by the ABC lab has focused on the P3 component of the ERP data collected. By focusing on the P3 component of ERP data rather than the mean ERP data alone, more specific results relating to the decision of MNCD could indicate significant markers to identify malingering individuals (Levada et al., 2016).

The latency analysis utilized MATLAB in combination with the extension EEGLAB and ERPLAB (Lopez-Calderon and Luck, 2014). Bilateral anterior and posterior sites were selected for analysis of fractional peak latency, which measures latency by finding the peak amplitude and then working backward in the waveform until 50% of that peak voltage is reached. Compared to simple peak latency, this is a better method that is optimal for finding onset latency and allows for most accurate results. The peak measures were tested for between group differences with a one-way ANOVA with a significance at the 0.05 level.

To further examine the implications of peak latency differences between groups, amplitudes from −200 to 600 ms at potentially significant electrodes were also examined. Significant electrodes areas were visualized from the development of scalp topographic maps of subjects over the same time frame. The topographic maps were created based on grand averaged data of all subjects within a testing group, done with MATLAB.

## Results

**FIGURE 2:**
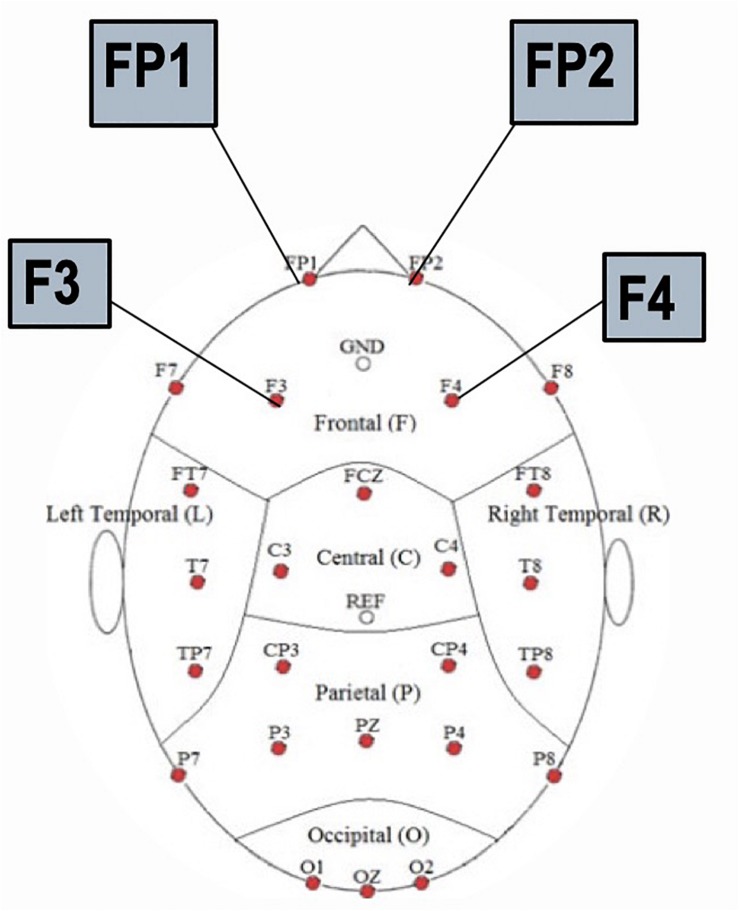
Locations of the frontal electrodes FP1, FP2, F3, and F4 on the 32 channel EEG cap.

**FIGURE 3:**
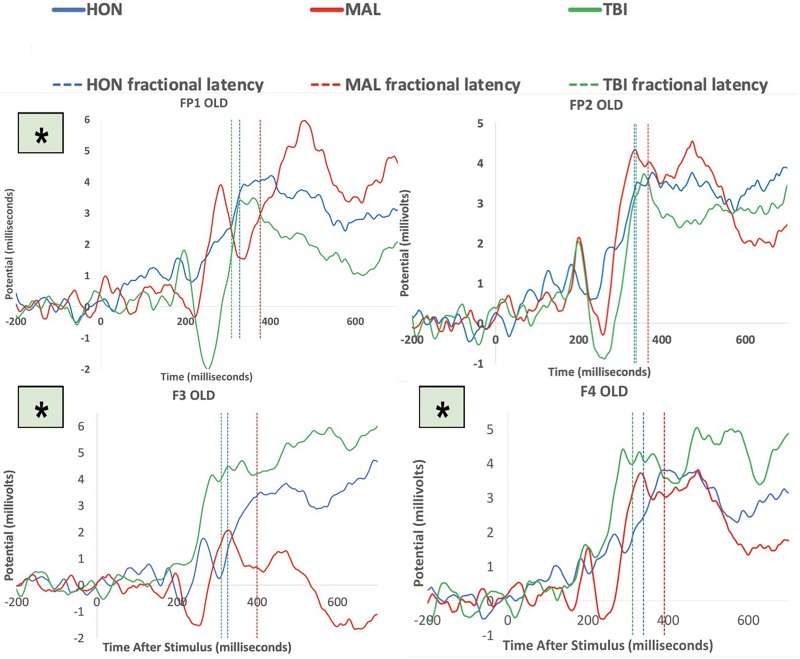
Group averaged responses during Old items at the electrodes FP1, FP2, F3, and F4. Solid lines represent grand average of ERP responses of honest subjects (blue), malingering subjects (red), and TBI subjects (green) at each electrode site. Dashed lines represent the averaged fractional peak latencies of each group with the same corresponding colors. Asterisks indicate significant results at the electrode. Note that all significant group differences between MAL and true TBI were during memory recognition of studied items.

To test the hypothesis of frontal manipulation among healthy individuals faking brain injury, we examined latencies of P3 in several bilateral frontal electrodes (i.e., FP1, FP2, F3, and F4; See Figure 2). Significant group differences were found with the Old (studied) memory recognition at these frontal electrodes. In using fractional peak latency analysis on these electrodes, the fractional peak latency for each subject group was compared to the grand averaged voltage data of brain activity over the −200 to 600 ms time frame for each group. This analysis allowed for visualization of the differences in peak latency between subject groups during Old (studied) memory recognition (Figure 3). As well as visualization, the data was tested for significance through a one-way ANOVA with a significance at the 0.05 level. The statistical analysis yielded results of significant differences in peak latency between MAL and TBI groups at the FP1, F3, and F4 electrodes for the Old condition only (Table 1). The largest latency differences between true TBI and malingerers are at the F3 site. The malingerers of memory deficits are on average 88 ms longer in the left frontal site. Latencies were also examined at occipital electrodes, but no significant group differences were found.

**TABLE 1:** The average fractional latency (ms) of bilateral frontal electrode sites.

**FP1**	**P3 latency (ms)**		**FP2**	**P3 latency (ms)**	
	**Old**	**New**		**Old**	**New**
HON	327.2 ± 56.9	344.0 ± 49.6	HON	332.8 ± 56.8	344.7 ± 48.5
MAL	375.7 ± 38.6^∗^	389.7 ± 28.7	MAL	366.6 ± 44.9	381.3 ± 34.5
TBI	307.8 ± 73.5^∗^	328.4 ± 71.9	TBI	337.3 ± 79.2	323.2 ± 63.3

**F3^∗^**	**P3 latency (ms)**		**F4**	**P3 latency (ms)**	
	**Old**	**New**		**Old**	**New**

HON	326.3 ± 57.6	369.7 ± 59.5	HON	338.0 ± 55.6	372.7 ± 56.1
MAL	397.5 ± 43.6^∗^	389.2 ± 35.1	MAL	390.1 ± 48.0^∗^	375.9 ± 44.1
TBI	309.4 ± 55.1^∗^	313.7 ± 77.4	TBI	310.9 ± 69.2^∗^	342.7 ± 74.3

*^∗^*p* < 0.05.*

**FIGURE 4:**
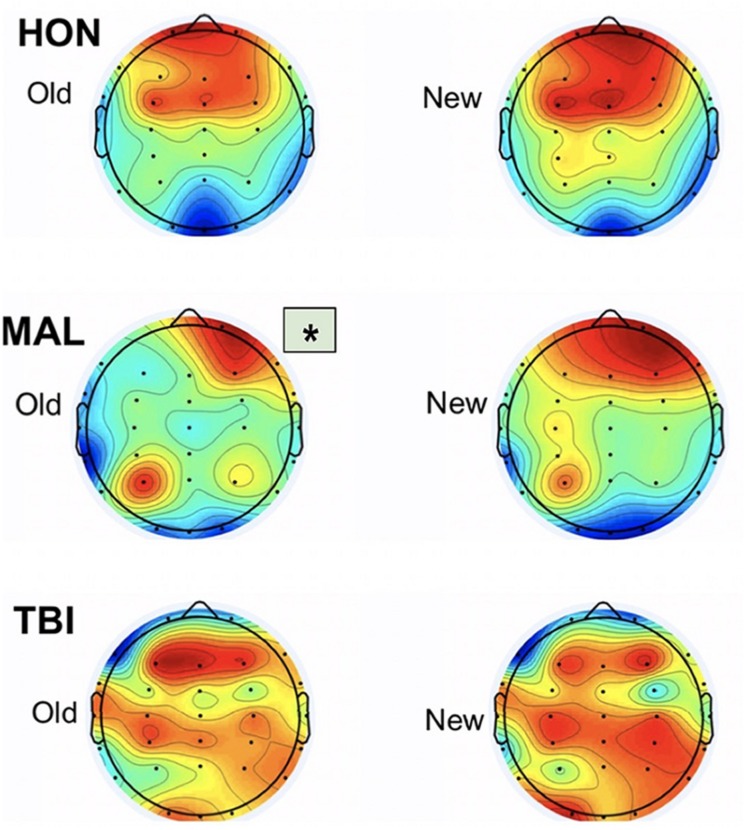
Topographic maps from the three testing groups representing averaged activity from 250 to 400 ms. Group differences were found only during old items. Asterisk indicates significant differences from other groups.

Topographic maps created with the resultant data indicated the brain regions with the highest average amplitude of activity during testing for subject groups, identified by dark red regions on the Figure 4. The information gathered from the topographic maps indicated that significant electrodes for analysis were located in the frontal and visual cortices as shown.

**FIGURE 5:**
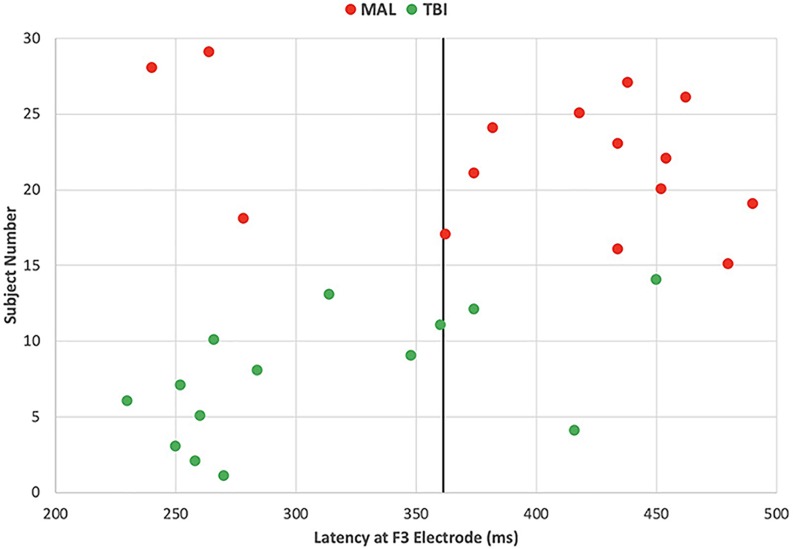
Individual’s latency at one left frontal site differentiates malingers with 80% sensitivity and 79% of specificity. Each point represents an individual subject’s P3 latency at a left frontal (F3) site, color coded by subject groups with red representing malingering and green representing brain injured individuals. The solid black line represents the delayed latency threshold at 361 ms.

The significance of the latency differences can also be visualized through scatter plot (Figure 5). Each group is represented by color (red = malingering, green = brain injured) with each point representing an individual subject. Honest subjects were not visualized in the figure because the aim is to differentiate malingerers from brain injured individuals. The solid black line at 361 ms represents the threshold of significant group differences in latency. The red points to the right of the line represent true positive values as they are delayed malingering latencies. The red marks to the left of the line represent false negative values as they are malingerers without significantly delayed latencies. The green marks to the left of the line represent the true negative values as they are traumatic brain injured individuals with no delay in latency. The green marks to the right of the line represent false positive values as they are brain injured individuals with delayed latencies. Using these values, the sensitivity or hit rate was calculated to be 80%, meaning that in using delayed latencies, 80% of malingering individuals will be positively identified. The specificity was also calculated with these values and found to be 79%.

## Discussion

We report new findings that the left frontal neural responses during recognition decision of studied visual stimuli are significantly delayed in malingerers compared to those in true patients with traumatic brain injured. The results indicate an averaged delay of 396 ms for malingerers compared to a 312 ms averaged delay for TBI individuals, marking an 84-ms difference in cognitive processing between the two groups. The results also indicate honest individuals using primarily bilateral frontal engagement when viewing both Old and New images. In contrast, malingering individuals engaged right frontal and left occipital regions in response to both New and Old images. Individuals with traumatic brain injuries engaged in distributed cortices: frontal, parietal, and mostly the right occipital visual cortex in response to both New and Old images. These differences in regional engagement between test groups are most evident from the scalp topographic maps and indicate notable differences in brain activity, not only between healthy and brain injured individuals, but also between individuals responding honestly to stimuli versus those malingering deficits.

The decision factors about ERP old/new effects was found to be associated with the late positive components (LPC) responses which had a left > right, centro-parietal scalp topography (Finnigan, 2002). Thus our analysis focused on lateralized electrodes. We found that only maligning group had asymmetrical frontal activity during their decision of whether to lie or not about an old-item. This frontal engagement might be the involvement of the working memory required to plan and exhibit TBI-like memory failure. Interestingly, during a modified delayed match-to-sample task, left-frontal memory related potentials during the working memory task discriminated healthy older adults and those with mild cognitive impairment patients. The LPC in the right frontal ERPs were statistically identical between normal older adults and those with early Alzheimers’ disease (Li et al., 2017). Fletcher and Henson (2001) determined there are two types of working memory tasks: “delayed matching tasks” and “self-ordered tasks.” The faking/fringing TBI process may require a malinger to determine if a probe stimulus matches a stimulus held in their memory similar to Old/New task in the experiment and then a self-ordered task to be honest or not for this item (Fletcher and Henson, 2001). Both the honest and TBI individuals performed to the best of their ability and merely answered whether they had seen the image previously. In contrast, the malingerers’ responses required different brain engagement because of their conscious effort exhibit TBI-like behavior, which is more similar to the “self-ordered tasks.” According to Petrides, individuals performing “delayed matching tasks” show engagement in the ventrolateral frontal cortex, while those performing “self-ordered tasks” show engagement in the dorsolateral frontal cortex (Petrides, 1995). These differing areas of engagement show further distinction between the neural responses of malingerers to that of honest and TBI individuals.

The left occipital engagement illustrates the visual processing of the malingerers as they viewed the images presented and determined if they had seen it before (Sehatpour et al., 2008). This engagement differs from honest participants whose engagement was focused mainly frontal bilaterally implying that honest participants engaged frontal-occipital communications differently from the malingerers because they were attempting to determine the correct categorization of the image (Old/New) while malingerers were less concerned with accuracy and more so with exhibiting a TBI-like performance.

Our present findings demonstrate a simpler way to measure neural delay that is harder to fake, which may lead to better clinical identifications of true TBI individuals from those who malinger deficits. The ERP results illustrate that the Old/New Memory task can provide clinicians with distinguishable markers in brain activity to differentiate malingerers from those with legitimate TBI. Although this form of testing is not immediate and requires the subject to perform the memory task, it yields quantifiable results to accurately identify TBI individuals and allow for them to get proper treatment without concern of exaggeration or malingering.

Although the results of analysis of P3 signatures found promising results, research has found that splitting the P3 signature into two components, P3a and P3b, could yield results more tailored to specific events (Polich, 2007). The P3a component deals specifically with detection of a stimulus, an involuntary response, while the P3b response is the conscious task-relevant processing of the stimulus (Hoover et al., 2014). Our results are consistent with the P3b component. In isolating specifically, the P3a component, comparisons can be made between malingerers and TBI individuals that can identify malingerers. Individuals attempting to malinger cognitive impairment could not simulate comparable P3a deficits seen in those with legitimate TBI. Abnormalities of these components may signal other mental disorders (Hoover et al., 2014; Bachiller et al., 2015). Because P3a is an involuntary reaction, differences in this component specifically can be a promising identifier of malingerers.

The findings are promising, but there are several limitations to the research. First, cross-validations with independent samples are important for this type of application. Second, the current method is limited in differentiating a liar from a TBI patient, or from healthy honest, but it is not clinical diagnosis test in TBI patient. Also, the sample size of each group (12–15 individuals) is small and presents problems in attempts to generalize these results to a larger population without significant effect size. Additionally, the sample size is too small to examine sex differences in brain responses during decision-making.

Recent advancement of EEG recording makes EEG screening wireless and easier to use in the clinics. Technological advances have made the use of EEG testing and ERP analysis more accessible in clinical settings. These results illustrate the possibilities of the use of ERP analysis in TBI vetting for future studies. This experiment is the early stages of more promising and expansive results. New, independent sampling and data collection is needed to further validate these findings and achieve concrete predictive values for those with TBI and those who malinger.

In practice, identification of those malingering deficits of TBI can be useful for not only healthcare professionals, but also those involved in insurance and legal processing. Vallabhajosula (2015) discusses the implications of employing neuroscience in criminal law, specifically detailing malingering and its assessment. Malingering can have legal implications where people are able to lie or exaggerate symptoms to avoid criminal conviction or military service. It is difficult for legal professionals to identify malingerers without proof because they can be accused of defamation by the potential malingerer (Weiss and Van Dell, 2017). The consequences of malingering are great; for military settings, those malingering deficits of injury or disability to avoid military service are subject to court-marshal and punishment (Malingering 83 U. S. C. §. 883, 2016). The promising findings of definitive methods of identification of malingerers can have great use to identify those malingering deficits to avoid legal responsibilities. With effective testing allowing for differentiation between TBI individuals and those malingering deficits, neurological signatures identified through research can help identify dishonest individuals. These techniques can be put into practice in court proceedings to distinguish honest individuals from those providing false testimony.

The present results contribute to future studies in developing combined methods of differentiation between TBI individuals and malingerers. For instance, machine learning type of classifications applying frontal latencies, frontal-parietal amplitudes and task performance (accuracy and reaction times) will greatly improve the precision. Previous study has found that larger P3 amplitudes correlate to faster behavioral responses, but peak amplitude latencies do not differ for behavioral reaction times (Ramchurn et al., 2014). Using fractional peak latency to compare the P3 signatures to reaction times could yield promising results. This method could allow for another form identification of possible differences in behavioral markers during memory tasks that can differentiate test groups. Research could also be useful in exploring more detailed identification of TBI to differentiate those with mild TBI versus those with severe. The Glasgow Coma Scale was developed to determine the level of consciousness of a person after a TBI. On the scale, a score of 13–15 is classified as mild, 9–12 as moderate, and 8 or less as severe (Teasdale and Jennett, 1974). Categorizing the TBI participants in the study could yield more specific results based on the severity of their brain injury. Studies have found that mild TBI results in prolonged P3 latencies at central electrodes compared to healthy individuals (Nandrajog et al., 2017). In contrast, the results found in this study show that the moderate to severe TBI individuals have early onset peak P3 latencies compared to healthy individuals. Differences in peak latencies could indicate a discernable pattern in brain activity based on the severity of TBI. In using methods to better detail the extent of TBI, patients would be able to receive more appropriate and tailored care for their level of injury.

## Data Availability

The datasets generated for this study are available on request to the corresponding author.

## Author Contributions

JN was responsible for writing the manuscript and performed some data analysis and interpretation. SS was involved in the data analysis of the fractional latency and interpretation as well as helped with the writing. EB contributed to the data analysis and drafting the manuscript. PC was aided with data analysis and interpretation. BW was responsible for early data analysis and interpretation. VV designed and collected the clinical and EEG data as part of her Ph.D. dissertation. YJ contributed to the study design, EEG data collection, analysis, interpretation, and writing the manuscript.

## Conflict of Interest Statement

The authors declare that the research was conducted in the absence of any commercial or financial relationships that could be construed as a potential conflict of interest.
